# C-reactive protein, an early marker of community-acquired sepsis resolution: a multi-center prospective observational study

**DOI:** 10.1186/cc10313

**Published:** 2011-07-15

**Authors:** Pedro Póvoa, Armando M Teixeira-Pinto, António H Carneiro

**Affiliations:** 1 Polyvalent Intensive Care Unit, São Francisco Xavier Hospital, CHLO, Estrada do Forte do Alto do Duque, 1449-005 Lisbon, Portugal; 2 CEDOC, Faculty of Medical Sciences, New University of Lisbon, Campo dos Mártires da Pátria, 130, 1169-056 Lisbon, Portugal; 3 Department of Biostatistics and Medical Informatics, CINTESIS, Faculty of Medicine, University of Porto, Alameda Prof. Hernâni Monteiro, 4200-319 Porto, Portugal; 4 Santo António Hospital, Largo Prof. Abel Salazar, 4099-001 Porto, Portugal

## Abstract

**Introduction:**

C-reactive protein (CRP) has been shown to be a valuable marker in the diagnosis of infection and in monitoring its response to antibiotics. Our objective was to evaluate serial CRP measurements after prescription of antibiotics to describe the clinical course of Community-Acquired Sepsis admitted to intensive care units (ICU).

**Methods:**

During a 12-month period a multi-center, prospective, observational study was conducted, segregating adults with Community-Acquired Sepsis. Patients were followed-up during the first five ICU days, day of ICU discharge or death and hospital outcome. CRP-ratio was calculated in relation to Day 1 CRP concentration. Patients were classified according to the pattern of CRP-ratio response to antibiotics: fast response if Day 5 CRP-ratio was < 0.4, slow response if Day 5 CRP-ratio was between 0.4 and 0.8, and no response if Day 5 CRP-ratio was > 0.8. Comparison between survivors and non-survivors was performed.

**Results:**

A total of 891 patients (age 60 ± 17 yrs, hospital mortality 38%) were studied. There were no significant differences between the CRP of survivors and non-survivors until Day 2 of antibiotic therapy. On the following three days, CRP of survivors was significantly lower (*P *< 0.001). After adjusting for the Simplified Acute Physiology Score II and severity of sepsis, the CRP course was significantly associated with mortality (OR_CRP-ratio _= 1.03, confidence interval _95%_= (1.02, 1.04), *P *< 0.001). The hospital mortality of patients with fast response, slow response and no response patterns was 23%, 30% and 41%, respectively (*P *= 0.001). No responders had a significant increase on the odds of death (OR = 2.5, CI_95% _= (1.6, 4.0), *P *< 0.001) when compared with fast responders.

**Conclusions:**

Daily CRP measurements after antibiotic prescription were useful as early as Day 3 in identification of Community-Acquired Sepsis patients with poor outcome. The rate of CRP decline during the first five ICU days was markedly associated with prognosis. The identification of the pattern of CRP-ratio response was useful in the recognition of the individual clinical course.

## Introduction

Sepsis is a complex clinical syndrome that complicates severe infection and is characterized by systemic inflammation and widespread organ dysfunction [1,2]. Traditional clinical signs of sepsis, such as fever, tachycardia, tachypnea and leukocytosis, are quite sensitive but poorly specific of infection, occurring in a variety of non-infectious conditions [3,4].

In most cases, the assessment of infection response to antibiotics relies mostly on the evolution of the same criteria used for diagnosis [5-7]. Microbiological criteria are also of little help in the assessment of response, because of the time needed to obtain culture results, the interference of antibiotics on bacterial growth *in vitro *and possible difficulties in recollecting some microbiological samples.

These areas of uncertainty in the clinical decision-making process led investigators to look at the inflammatory cascade for potential objective markers of infection [8,9]. These biomarkers, among which C-reactive protein (CRP) is one of the most studied [10,11], could be used as surrogates of infection diagnosis.

It has been shown that a single CRP measurement helps in the diagnosis of infection [12-14] with some controversy concerning prognosis [15,16], and that serial determinations are useful in the prediction of infection [17,18] as well as in monitoring its response to treatment [19-23]. In addition, it has been described, in ventilator associated pneumonia (VAP), a good correlation between bacterial load and CRP levels [24], as well as between the adequacy of antibiotic therapy and changes of CRP concentration overtime [19,24-26]. Besides, serial CRP measurements enable clinicians to identify individual CRP patterns of response to antibiotic therapy with a good correlation with the individual clinical course [19,20,23,27]. Finally, a recent trial, performed in an outpatient setting, clearly demonstrated that the availability of CRP had a significant impact on antibiotic prescription [28].

Therefore, further investigation in this area is critical, assessing the course of biomarkers after prescription of antibiotic therapy in a larger cohort of infected intensive care unit (ICU) patients. The aim of the present study was to evaluate the value of serial CRP measurements after prescription of antibiotics to describe clinical course in patients with community-acquired sepsis (CAS) admitted in ICU.

## Materials and methods

### Design and setting

The SACiUCI study (*Sepsis Adquirida na Comunidade e internada em Unidade de Cuidados Intensivos*) was a prospective, multiple-center, observational study, designed to evaluate the epidemiology of CAS. Details of study design, definitions, data collection and management are provided elsewhere [29].

Briefly, all patients who were ≥ 18 years old and newly admitted with CAS to the participating ICU (see Additional file 1 for a list of the participating ICU) from December 2004 to November 2005 were consecutively enrolled. Almost two thirds of the patients with sepsis were admitted from the emergency room of the hospital of the participating ICU, and the remaining came from emergency departments of other hospitals. Patients were followed-up until death or hospital discharge. Only the first ICU admission was included. Since this observational study did not require any deviation from routine medical practice, Institutional Review Boards approved the study design and waived the need of informed consent.

### Definitions and selection of participants

Infection was defined as a pathologic process caused by the invasion of normally sterile tissue or fluid or body cavity by a pathogenic or potentially pathogenic microorganism [1] and/or clinically suspected infection, plus the prescription of antimicrobial therapy. Community-acquired infection was defined as the onset of infection prior to hospital admission or not present at admission but becomes evident in the first 48 hrs [30]. Presence of sepsis was defined according to the American College of Chest Physicians/Society of Critical Care Medicine Consensus Conference criteria [31].

The presence of health-care associated infection (HCAI) was defined at hospital admission according to the presence of the following criteria: home infusion therapy (including antibiotics) or home wound care; chronic dialysis or chemotherapy within 30 days; hospitalization for 2 days or more in the preceding 90 days; residence in a nursing home or extended care facility [32,33].

### Data collection

Data were collected prospectively either using pre-printed case report forms, a specific base software or on-line through the study web page.

Data collection included demographic data and comorbid diseases. Clinical and laboratory data at the time of hospital admission was recorded. The Simplified Acute Physiology Score (SAPS) II [34] was calculated. Microbiological and clinical infectious data were reported. C-reactive protein, body temperature, white blood cell count (WBC) and the Sequential Organ Failure Assessment (SOFA) score [35] were evaluated during the first five days of ICU stay. Blood samples were obtained from an arterial line at ICU admission and subsequently every morning.

For purposes of the time dependent analysis Day 1 (D1) was defined as the day of ICU admission. The following days were successively named as D2, D3 up to D5.

The withdrawal of the inflammatory stimulus results in a sharp decrease of the CRP serum concentration in a way similar to a first-order elimination kinetics [36,37]. Consequently, relative CRP variations are more informative that absolute changes. As a result we assessed a new variable, CRP-ratio, which was calculated in relation to D1 CRP concentration. Subsequently, we classified patients according to three patterns of CRP-ratio response to antibiotic therapy: fast reponse, if D5 CRP value was less than 40% of D1 value; slow response, if D5 CRP was between 40% and 80% of D1; and no response, if D5 CRP was higher than 80% of D1 [10,11,19]. The CRP-ratio patterns were a modification of others previously published [19,20,27].

The evolution of CRP, CRP-ratio, temperature and WBC during the five days of CAS course was analyzed comparing survivors and non-survivors.

### Data processing and statistical analysis

Data entry was performed by a single investigator in each participating center and consistency was assessed with a rechecking procedure of a 10% random sample of patients (see Acknowledgements). Data were screened in detail (see Acknowledgements) for missing information, implausible and outlying values.

Continuous variables were expressed as means and standard deviations (SD) or median and interquartile range (IQR) if the distribution was clearly assymetric. Comparisons between groups were performed with two-tailed unpaired Student's *t*-test, one-way ANOVA, Mann-Whitney *U *or Kruskal-Wallis *H *tests for continuous variables according to data distribution. Fisher's exact test and Chi-square test was used to carry out comparisons between categorical variables as appropriate.

**Figure 1:**
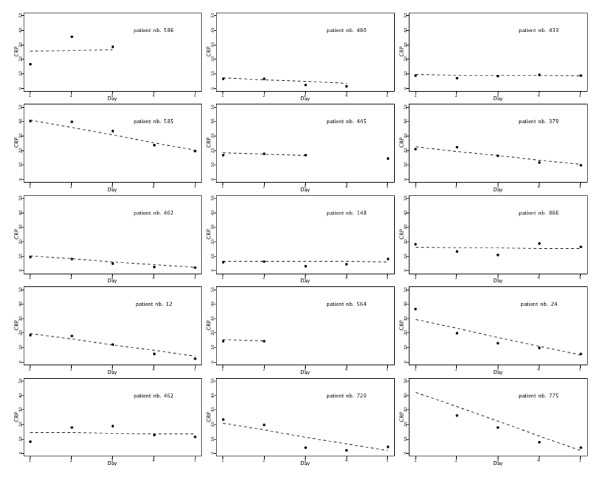
**Individual CRP course for a group of patients during the first five ICU days**. Dots indicate the individual observed values at the different time points, from day 1 to day 5, and the dashed lines represent the predictions obtained by a linear mixed model for CRP with random intercept and random slope for time. The intercept (α) describes the initial CRP value and the slope (β) describes the CRP rate of change per day for a specific patient (see text for further explanation). CRP, C-reactive protein; ICU, intensive care unit.

For the statistical analysis of the patient's status at hospital discharge as function of a longitudinal covariate (five measurements of CPR) we used a two-step approach. First, we modeled the CRP measurements as a function of time (five days of CRP measurement). We used a linear mixed model that allowed us to predict an intercept and a slope for each patient. This step is conceptually similar to fitting a linear regression, Mean_CRP = α + β*day, for each patient and obtaining an individual intercept (α) and slope (β) per patient. The *intercept *describes the initial CRP value and the *slope *describes the CPR rate of change per day for a specific patient. We assumed a normal distribution of the random effects. Once the model was fitted, the estimates for the intercepts and slopes were obtained as the best linear unbiased predictors, BLUPs [38]. Figure 1 shows the observed values of CRP and predicted values by the model for a randomly selected group of patients.

For the second step, we started by computing the relative rate of CRP change, CRP-ratio, per day for each patient which is obtained by dividing the slopes by the intercepts. This quantity gives the percentage of change per day in CRP, relative to the first measurement (intercept). Then, we used the individual CRP-ratios and intercepts, together with SAPS II and sepsis severity (uncomplicated sepsis, severe sepsis and septic shock), as covariates in a logistic regression for hospital mortality. This model allowed us to compute the odds ratios (OR) associated with the CRP profile (expressed as an intercept and a rate of change per day, the CRP-ratio) adjusted for patient's severity (SAPS II and sepsis severity). Because the covariates "intercept" and "CRP-ratio" are estimates and not observed values, the standard errors provided by the software are not correctly computed. Thus, we obtained estimated of the correct standard errors for the regression coefficients through bootstraping. A similar approach was used for the five days of measurements of temperature and WBC. However, WBC was skewed and we used a transformation by taking the natural log of WBC (log-WBC) to comply with the normal assumption.

Additionally, we analysed the association between hospital mortality and CRP by considering the patterns of CRP-ratio response. Only patients with available measurements at D1 and D5 were considered for this analysis. We computed the OR of hospital mortality for the CRP-ratio patterns using a logistic regression and adjusting for SAPS II and sepsis severity.

For all logistic models, we checked the Hosmer and Lemeshow goodness-of-fit and computed the receiver operating characteristic (ROC) curve to evaluate model discrimination. Data were analyzed using PASW v.18.0 for MAC (SPSS, Chicago, IL, USA) and R (Development Core Team. R: A Language and Environment for Statistical Computing. Vienna, Austria: 2005). All statistics were two-tailed and significance level was set at 0.05.

## Results

### Characteristics of the study population

A total of 891 patients with CAS were included (Table 1); 209 patients were classified as having HCAI. The lungs were the most common site of infection (61%), followed by the abdomen (18%) and urinary tract (7%). Cultures were positive in 40% of the patients, with *Streptococcus pneumoniae *(21%), *Escherichia coli *(18%) and methicillin sensitive *Staphylococcus aureus *(12%) the three most common isolated microorganisms. On ICU admission, 9% of CAS patients presented with uncomplicated sepsis, 40% severe sepsis and 51% septic shock. The overall ICU and hospital mortality rate of CAS was 30% (N = 265) and 38% (N = 338), respectively.

**Table 1:** Baseline characteristics of community-acquired sepsis patients

Characteristic^a^	Total	Survivors	Nonsurvivors	*P*
	N = 891	N = 553	N = 338	
Age, mean ± SD	60 ± 17	58 ± 18	65 ± 16	< 0.001
Male sex, n (%)	574 (64%)	344 (60%)	230 (40%)	0.071^b^
SAPS II, mean ± SD	50 ± 19	44 ± 15	60 ± 20	< 0.001
SOFA, mean ± SD	7.4 ± 3.0	6.3 ± 3.3	9.1 ± 4.0	< 0.001
Mechanical ventilation, n (%)	820 (92%)	487 (88%)	335 (99%)	< 0.001
RRT, n (%)	62 (7%)	33 (6%)	30 (9%)	0.043
Primary admission diagnoses, n (%)				
Medical non coronary	693 (78%)	423 (61%)	270 (39%)	0.023
Medical coronary	10 (1%)	3 (30%)	7 (70%)	
Trauma	38 (4%)	31 (82%)	7 (18%)	
Scheduled surgery	4 (< 1%)	2 (50%)	2 (50%)	
Emergency surgery	146 (16%)	94 (64%)	52 (36%)	
CRP (mg/dl), mean ± SD	20.1 ± 13.9	19.8 ± 12.5	20.7 ± 12.8	0.367
Temperature (°C), mean ± SD	37.5 ± 1.2	37.5 ± 1.3	37.5 ± 1.1	0.799
WBC (×10^3^/mm^3^), median (IQR)	12.4 (8 to 19)	12.4 (9 to 19)	12.4 (7 to 19)	0.496
ICU LOS, days, median (IQR)	9 (5 to 15)	9 (6 to 14)	8 (3 to 17)	< 0.001^c^
Hospital LOS, days, median (IQR)	18 (10 to 29)	21 (14 to 31)	11 (4 to 23)	< 0.001^c^
Severity of sepsis				
Uncomplicated sepsis	83 (9%)	67 (81%)	16 (19%)	< 0.001
Severe sepsis	358 (40%)	265 (74%)	93 (26%)	
Septic shock	450 (51%)	221 (49%)	229 (51%)	

^a ^CRP, C-reactive protein; ICU, intensive care unit; IQR, interquartile range; LOS, length of stay; NS, not significant; SAPS, Simplified Acute Physiology Score; SD, standard deviation; SOFA, Sequential Organ Failure Assessment; RRT, renal replacement therapy; WBC, white blood cell count.

^b ^Fisher's exact test

^c ^Mann-Whitney *U *test

### C-reactive protein as a marker of sepsis resolution

**Figure 2:**
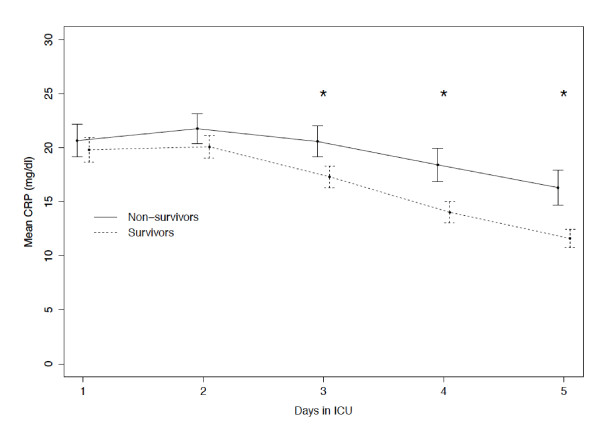
**CRP course during the first five ICU days in survivors and nonsurvivors**. Observed means of CRP during the first five days in ICU stay for survivors (dashed line) and nonsurvivors (solid line) at hospital discharge. Error bars represent point-wise 95% confidence intervals. (*P < 0.001); CRP, C-reactive protein; ICU, intensive care unit.

At D1 (Table 1), CRP of survivors and non-survivors was not statistically different, 19.8 ± 12.5 mg/dL vs. 20.7 ± 12.8 mg/dL (*P *= 0.367). When we compared CRP (Figure 2) of survivors and non-survivors at the different time points, we found that CRP of non-survivors was significantly higher since D3 onwards (*P *< 0.001, for D3, D4 and D5).

After adjusting for SAPS II and the severity of sepsis, the initial value of CRP remained not significantly associated with hospital mortality (OR_initial _= 1.01, confidence interval (CI)_95% _= (0.99, 1.02), *P *= 0.297). On the contrary, the course of CRP measured as the relative changes during the first five ICU days, was significantly associated with hospital mortality (OR_CRP-ratio_= 1.03, CI_95% _= (1.02, 1.04), *P *< 0.001). For example, a patient with an average decrease of CRP concentration of 10% per day has 32% less chances of dying when compared to a patient with the same SAPS II score and the same severity of sepsis but with no CRP decrease. The ability of the model to predict hospital outcome assessed by the area under the ROC curve was 0.77 (CI_95% _= (0.73; 0.80)).

To assess the impact of the variable HCAI, we added this variable to our model to study its interaction with the initial value of CRP and CRP-ratio. The interactions were not significant (*P *= 0.579 and *P *= 0.182, respectively), indicating that there is no evidence in our patient population of a different behaviour of CRP for this specific subgroup of septic patients.

The same analysis, restricted to the subgroup of septic shock patients, presented similar results (data not shown).

### Temperature and WBC and resolution of sepsis

At D1, temperature and WBC of survivors and non-survivors were not statistically different (Table 1), 37.5 ± 1.3°C vs. 37.5 ± 1.1°C (*P *= 0.799) and median (IQR) 12.4 (8.5, 18.7) × 10^3^/mm^3 ^vs. 12.4 (6.9, 18.5) × 10^3^/mm^3 ^(*P *= 0.496), respectively. Time dependent analysis of temperature from D1 to D5 showed no association between temperature evolution and survival (*P *= 0.360). Temperature decreased likewise in survivors and non-survivors. Similarly, no differences were found on the WBC course along the first five days of the ICU stay (*P *= 0.594).

### Clinical course and sepsis resolution

Sepsis resolution during antibiotic therapy was also monitored with daily measurements of SOFA score. The time dependent analysis of SOFA score from D1 to D5 of antibiotic therapy of survivors and nonsurvivors was significantly different (*P *< 0.001). In survivors the SOFA decreased steadily from 6.3 ± 3.3 at D1 to 4.9 ± 3.3 at D5 whereas in non-survivors it remained roughly unchanged (D1: 9.1 ± 4.0 and D5: 9.0 ± 4.0).

### Patterns of CRP-ratio response to antibiotics

Community-acquired sepsis patients were divided according to three patterns of CRP-ratio response to antibiotic therapy. Two hundred died or were discharged before D5. One hundred and thirty five patients had no CRP recorded at D1 or D5. Five hundred, fifty-six patients had complete data for the analysis. One hundred, ninety-nine (36%) patients were classified as fast response, 149 (27%) as slow response and 208 (37%) as no response.

The ICU mortality rate was significantly different according to the patterns of CRP-ratio response: fast response 14%, slow response 20% and no response 32% (*P *< 0.001). After adjusting for SAPS II and severity of sepsis, the odds of death for slow or no response patients were 1.6 (CI_95% _(0.9, 2.9), *P *= 0.119) and 3.0 (CI_95% _(1.8, 5.1), *P *< 0.001), respectively, when compared with the fast response ones. By D3, median CRP-ratio (5^th ^and 95^th ^percentiles) was 0.81 (0.40, 1.30), 0.95 (0.62, 1.48) and 1.22 (0.70, 6.64) in patients with fast response, slow response, no response patterns, respectively (*P *< 0.001).

The hospital mortality was also significantly associated with the pattern of response (*P *= 0.001). Fast responders have lower mortality (23%) when compared with slow and no responders (30% and 41%, respectively). After adjusting for SAPS II and severity of sepsis, the pattern of CRP-ratio response remained significantly associated with mortality. No responders had a significantly increase on the odds of mortality (OR = 2.5, CI_95% _= (1.6, 4.0), *P *< 0.001) when compared with fast responders. Slow responders showed a non-significant increase on the odds of mortality in comparison with the fast responders (OR = 1.5, CI_95% _= (0.9, 2.5), *P *= 0.124).

The same analysis, restricted to the subgroup of septic shock patients, presented similar results (data not shown).

### Association between CRP-ratio patterns of response with temperature and WBC course

**Figure 3:**
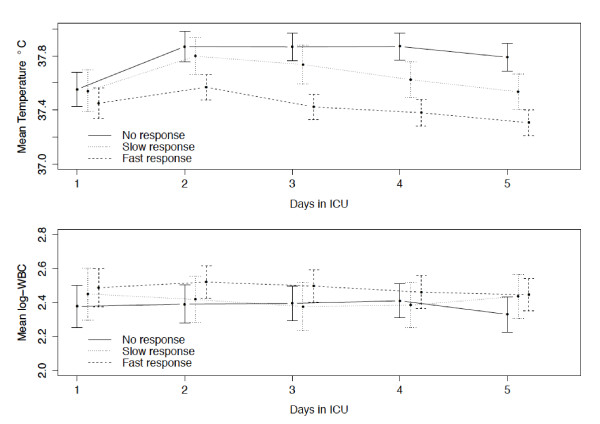
**Temperature and WBC course during the first five ICU days according to CRP-ratio patterns**. Mean temperature and WBC count (log transformed) during the first five days in ICU, according to the different patterns of CRP-ratio response to antibiotics. Error bars represent point-wise 95% confidence intervals. CRP, C-reactive protein; ICU, intensive care unit; WBC, white blood cell.

Temperature decreased significantly faster for the fast and slow response patterns groups when compared with the no response pattern group (*P *= 0.004 and *P *< 0.001, respectively) (Figure 3). However, the D1 and D5 temperature of patients with the fast and slow response CRP-ratio patterns was very similar (D1: 37.4 ± 1.1°C and 37.5 ± 1.0°C and D5: 37.3 ± 0.8°C and 37.5 ± 0.9°C for fast and slow response, respectively), while the temperature of the patients with no response CRP-ratio pattern increased slightly from 37.6 ± 1.1°C at D1 to 37.8 ± 0.9°C at D5.

On the other side, no significant time trend was observed regarding WBC for the fast response pattern (*P *= 0.137) and no significant differences were found for the slow and no response pattern groups (*P *= 0.680 and *P *= 0.619, respectively) in comparison to the fast response group (Figure 3).

### Association of CRP-ratio patterns of response with clinical course

**Figure 4:**
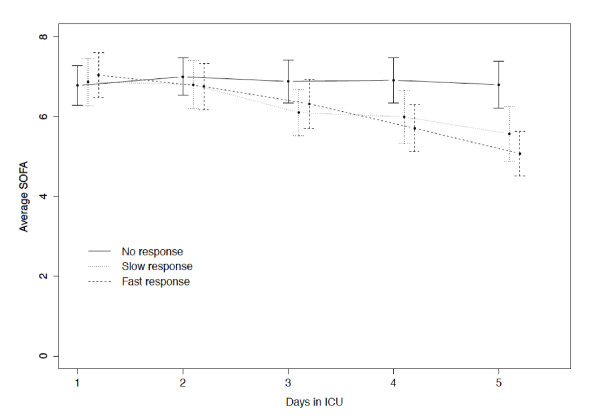
**SOFA score course during the first five ICU days according to CRP-ratio patterns**. Mean SOFA score for the first five days in ICU stay, according to the patterns of CRP-ratio response to antibiotics: fast response, slow response and no response. Error bars represent point-wise 95% confidence intervals. CRP, C-reactive protein; ICU; intensive care unit; SOFA, Sequential Organ Failure Assessment.

The time dependent analysis of the SOFA score of the three patterns of CRP-ratio response from D1 to D5 showed that these patterns of evolution were significantly different (fast vs. slow responders, *P *= 0.003 and fast vs. no responders, *P *< 0.001) (Figure 4). The SOFA scores of patients with the fast and slow CRP-ratio response patterns decreased from 7.0 ± 3.7 and 6.9 ± 3.4 at D1 to 5.1 ± 3.6 and 5.6 ± 3.9 at D5. Opposed to this were the SOFA scores of the patients with no response, in which the CRP-ratio pattern remained almost unchanged, from 6.8 ± 3.3 at D1 to 6.8 ± 3.9 at D5.

## Discussion

In the present study, we assessed the course of serum CRP concentrations in the first five days after prescription of antibiotics in a large cohort of CAS patients admitted to ICU. In survivors, CRP showed a marked decrease and was significantly lower in comparison to non-survivors from D3 onwards. Besides, the identification of an individual pattern of CRP-ratio response showed that patients with a no response pattern had more than three times the odds of dying in the ICU when compared to patients with a fast response pattern. Based on our findings, daily CRP measurements can be used as an early marker of CAS resolution and might be helpful in the clinical-decision making process, since the pattern of CRP-ratio response correlates with the individual clinical course, improving or worsening, as well as, the rate of improvement.

The criteria used to assess infection response to antibiotics [6,7] can be influenced by several non-infectious factors [19]. Consequently, the reliance on those markers, not only make the diagnosis of sepsis but also the evaluation of the response to therapy often misleading.

Even though the Clinical Pulmonary Infection Score (CPIS) [39] was not designed to monitor VAP response to antibiotics, Luna *et al. *[40] studied the performance of serial CPIS measurements in VAP resolution. In comparison with non-survivors, CPIS of survivors was significantly lower at D3 and D5, but not at D7. However, others did not reproduce these findings [41].

These limitations led investigators to look at the inflammatory cascade for potential objective markers of infection [8]. Many serum markers have been assessed in sepsis [2,42]; procalcitonin and CRP being the more extensively studied [11,43].

We have already shown in patients with VAP [19], bloodstream infections [20] and community-acquired pneumonia [27], that in survivors CRP decreases in the first days of antibiotic therapy while in non-survivors it remains almost unchanged. In the SACiUCI study, we reproduced those findings in a much larger cohort of CAS patients, clearly showing that survivors present an early decrease in CRP concentration that was significantly lower than that of non-survivors, from D3 onwards (*P *< 0.001). Taking into account data from other studies [19,23-27], it seems that CRP could be used as an early marker, as early as D3, of infection response to antibiotics. Since early CRP changes correlate with the adequacy of antibiotic therapy [19,24-26] and to the bacterial load, at least in VAP patients [24], it seems that the CRP course could be used as a surrogate marker of the clinical course of infection [11].

We have also previously introduced the concept of patterns of CRP-ratio response to antibiotic therapy [19,20,27]. In the SACiUCI study, with a much larger sample size, we have reproduced the preliminary findings, demonstrating that patients with fast and slow response CRP-ratio patterns had a significantly lower mortality than those in which CRP-ratio remained elevated.

We went further in our analysis to study the association between CRP-ratio patterns of response with the evolution of the SOFA score. At D1, patients with different patterns of CRP-ratio response showed a similar SOFA score. However, patients with fast and slow response patterns showed an almost parallel and significant decrease in SOFA score. In contrast, in patients with no response pattern SOFA score remained almost unchanged over the study period. This comparable evolution between the CRP-ratio course and SOFA score was also observed in survivors and non-survivors. In other words, increasing or persistently high CRP concentrations, suggest persistence of inflammatory activity, and were associated with poor prognosis, whereas decreasing CRP levels suggests resolution of the inflammatory response, and was associated with a better outcome [15].

Our study has several important strengths. To date, as far as we are aware, this is the largest multiple-center study evaluating the course of a biomarker, CRP, over a period of five days that are the crucial days in which to assess an infection response. The majority of included patients were admitted with severe sepsis and septic shock and all eligible patients were included, without exclusions, independently of the presence of comorbities, previous antibiotic therapy, underlying diagnosis or bacteriologic isolates that make our study population more representative of the current clinical practice.

However, we recognise that our study also has limitations. The nonrandomized and observational nature of the study design bears the potential of unmeasured confounders that may have caused differences in therapeutic and supportive approach, namely initiation of antibiotic therapy. We studied a mixed group of medical and surgical CAS patients. It is possible that other types of patients (for example, solid organ transplant recipients, febrile neutropenia, allogeneic stem cell transplantation) as well as infections (for example, viral infections) may have different CRP time-courses. Finally, we only assessed CRP. It is possible that other biomarkers, namely procalcitonin, might have different results.

## Conclusions

In conclusion, in severe CAS, daily CRP measurement during the first five ICU days after antibiotic prescription was useful in the identification, as early as D3, of patients with poor outcome. The rate of CRP decrease, assessed by the CRP-ratio course, was markedly associated with clinical course and prognosis. In addition, the identification of the pattern of CRP-ratio response to antibiotic therapy was useful in the recognition of the individual clinical evolution of CAS patients, improving or worsening, as well as, of the rate of improvement. With the present data, as well as from previously published papers assessing CRP monitoring, we think it is time to safely design a trial to assess the impact of the CRP-guided antibiotic therapy approach, not only to shorten antibiotic therapy but also to identify earlier patients not responding to treatment.

## Key messages

• In this large multicenter prospective observational study, we showed that daily CRP measurement after antibiotic prescription was useful, as early as Day 3, in discriminating community-acquired sepsis patients with good and bad outcome.

• The rate of CRP decrease, assessed by the CRP-ratio course, was markedly associated with clinical course and prognosis; a patient with an average decrease of the CRP of 10% per day has 32% more chances of surviving.

• We identified three patterns of CRP-ratio response to antibiotics, fast response, slow response and no response, with a marked correlation with ICU and hospital mortality.

• The identification of CRP-ratio pattern of response to antibiotics was useful in the recognition of the individual clinical course of community-acquired sepsis patients, improving or worsening, as well as, of the rate of improvement; a patient with a no response pattern has 3.0 times more chances of dying in the ICU in comparison with a patient with fast response.

## Abbreviations

BLUPs: best linear unbiased predictors; CAS: community-acquired sepsis; CI: confidence interval; CPIS: Clinical Pulmonary Infection Score; CRP: C-reactive protein; HCAI: health-care associated infection; ICU: intensive care unit; IQR: interquartile range; LOS: length of stay; OD: odds ratio; ROC: receiver operating characteristic; SAPS: Simplified Acute Physiology Score; SD: standard deviation; SOFA: Sequential Organ Failure Assessment; VAP: ventilator associated pneumonia; WBC: white blood cell.

## Competing interests

PP has received honoraria and served as advisor of Astra Zeneca, Ely-Lilly, Gilead, Janssen-Cilag, Merck Sharp & Dohme, Novartis and Pfizer and received unrestricted research grants from Brahms and Virogates. AMT-P has no competing interests to declare. AHC has no competing interests to declare.

## Authors' contributions

PP contributed to the study conception and design, carried out and participated in data analysis and drafted the manuscript. AMT-P carried out and supervised data analysis and drafted the manuscript. AHC conceived the study, participated in its design and coordination, participated in data analysis and helped to draft the manuscript. All authors read and approved the final manuscript.

## Authors' information

PP is Director of the Polyvalent Intensive Care Unit of São Francisco Xavier Hospital. PP is Professor of Medicine of the Faculty of Medical Sciences from the New University of Lisbon, Portugal. AMT-P is Professor of Biostatistics of the Faculty of Medicine from the University of Porto, Portugal. AHC was previously Director of the Polyvalent Intensive Care Unit of the Santo António Hospital, Porto, Portugal. Currently AHC is Director of the Department of Medicine, Emergency and Intensive Care of Arrábida Hospital, Gaia, Portugal.

## Supplementary Material

Additional file 1 **List of participating institutions**. Click here for file
