# Supplementary material for: C-reactive protein, an early marker of community-acquired sepsis resolution: a multi-center prospective observational study
Source: Crit Care. 2011 Jul 15;15(4):R169. doi: 10.1186/cc10313 (PMC3387609; doi:10.1186/cc10313)
Supplement: Additional file 1 — List of participating institutions. [file cc10313-S1.DOC]

**List of the participants in the SACiUCI study**

*Study coordinator*: António H. Carneiro (Unidade de Cuidados Intensivos Polivalente, Hospital Geral de Santo António, Porto)

*Study promoters*: António H. Carneiro (Unidade de Cuidados Intensivos Polivalente, Hospital Geral de Santo António, Porto), Eduardo Silva (Unidade de Cuidados Intensivos Polivalente, Hospital do Desterro, Lisboa) and José Artur Paiva (Unidade de Cuidados Intensivos Polivalente da Urgência, Hospital de São João, Porto)

*Participating Intensive Care Units*: Unidade de Cuidados Intensivos Polivalente, Hospital Geral de Santo António, Porto (Teresa Cardoso), Unidade de Cuidados Intensivos Polivalente da Urgência, Hospital de São João, Porto (José Manuel Pereira), Unidade de Cuidados Intensivos, Instituto Português de Oncologia, Porto (Filomena Faria), Unidade de Cuidados Intensivos Médicos, Hospital Pedro Hispano, Matosinhos (António Sarmento, Luís Ribeiro), Unidade de Cuidados Intensivos, Centro Hospitalar de Vila Nova de Gaia, Gaia (Paula Castelões), Unidade de Cuidados Intensivos, Hospital da Senhora da Oliveira, Guimarães (Anabela Bártolo), Unidade de Cuidados Intensivos, Hospital de São Pedro, Vila Real (Francisco Esteves), Unidade de Cuidados Intensivos, Hospital de São Sebastião, Santa Maria da Feira (Piedade Amaro), Serviço de Medicina Intensiva, Hospitais da Universidade de Coimbra, Coimbra (Paulo Martins), Unidade de Cuidados Intensivos, Centro Hospitalar de Coimbra, Coimbra (Paula Coutinho), Unidade de Cuidados Intensivos Polivalente, Hospital do Desterro, Lisboa (Paulo Gomes), Serviço de Medicina Intensiva, Hospital de Santa Maria, Lisboa (Henrique Bento), Unidade de Urgência Médica, Hospital de São José, Lisboa (Luís Reis), Unidade de Cuidados Intensivos Médicos, Hospital de São Francisco Xavier, Lisboa (Pedro Póvoa), Unidade de Cuidados Intensivos Médico-Cirúrgica, Hospital Pulido Valente, Lisboa (Armindo Ramos), Unidade de Cuidados Intensivos, Hospital José Joaquim Fernandes, Beja (José Vaz), Unidade de Cuidados Intensivos, Centro Hospitalar do Barlavento Algarvio, Portimão (Carlos Glória).
